# Autofocus Entropy Repositioning Method Bioinspired in the Magnetic Field Memory of the Bees Applied to Pollination

**DOI:** 10.3390/s21186198

**Published:** 2021-09-16

**Authors:** Daniel de Matos Luna dos Santos, Ewaldo Eder Carvalho Santana, Paulo Fernandes da Silva Junior, Jonathan Araujo Queiroz, João Viana da Fonseca Neto, Allan Kardec Barros, Carlos Augusto de Moraes Cruz, Viviane S. de Aquino, Luís S. O. de Castro, Raimundo Carlos Silvério Freire, Paulo Henrique da Fonseca Silva

**Affiliations:** 1 Graduating Program in Electrical Engineering, Federal University of Maranhão, Sao Luis 65085-580, Brazil; queirozjth@gmail.com (J.A.Q.); joao.fonseca@ufma.br (J.V.d.F.N.); akduailibe@gmail.com (A.K.B.); 2 Gradutation Program in Computation Engineering and Systems, State University of Maranhão, Sao Luis 65081-000, Brazil; ewaldosantana@professor.uema.br (E.E.C.S.); paulo.junior@ee.ufcg.edu.br (P.F.d.S.J.); 3 Graduation Program in Electrical Engineering, Federal University of Amazonas, Manaus 69080-900, Brazil; carlosamcruz@ufam.edu.br (C.A.d.M.C.); vivisaquino@gmail.com (V.S.d.A.); luisfne@gmail.com (L.S.O.d.C.); 4 Electrical Engineering Department, Federal University of Campina Grande, Campina Grande 58019-900, Brazil; rcsfreire@dee.ufcg.edu.br; 5 Coordination of PostGraduate Studies in Electrical Enginnering, Federal Institute of Paraíba, Joao Pessoa 58059-900, Brazil; phdafs@gmail.com

**Keywords:** bioinspired method, unsupervised repositioning, precision agriculture

## Abstract

In this paper, a bioinspired method in the magnetic field memory of the bees, applied in a rover of precision pollination, is presented. The method calculates sharpness features by entropy and variance of the Laplacian of images segmented by color in the HSV system in real-time. A complementary positioning method based on area feature extraction between active markers was developed, analyzing color characteristics, noise, and vibrations of the probe in time and frequency, through the lateral image of the probe. From the observed results, it can be seen that the unsupervised method does not require previous calibration of target dimensions, histogram, and distances involved in positioning. The algorithm showed less sensitivity in the extraction of sharpness characteristics regarding the number of edges and greater sensitivity to the gradient, allowing unforeseen operation scenarios, even in small sharpness variations, and robust response to variance local, temporal, and geophysical of the magnetic declination, not needing luminosity after scanning, with the two freedom of degrees of the rotation.

## 1. Introduction

According to the Union Nations, the world population shall be around 9.7 billion people in 2050 [1]. This directly implies that there will be a higher demand for food, and the use of better cultivation techniques by automation in agriculture, by the use of sensors, optimization of the robots, and computational systems, seeking to increase productivity and concepts related to precision agriculture.

According to [2], precision agriculture comprises the use of optimized resources to generate larger production per hectare, considering the geographical positioning, gathering information, and decision support, substantiated by the analysis of the environment variables by computation software.

Among the equipment employed, the rovers connected by wireless sensors’ networks are important for the agricultural automating processes related to soil and plants, with parameter analysis of complex agricultural processes. The analysis of data from these systems shows that productivity improvement is possible through the application of artificial pollination of flowers, harvesting, plowing, and soil irrigation, among others [2,3,4,5,6].

Rovers used in pollination are electromechanical systems composed of mechanisms with one or more sensors and actuators embedded. They are employed to perform data acquisition and decision making, with movement control over four degrees of freedom [7]. The use of bioinspired solutions is able to increase the efficiency of the rover’s design, enabling thus the reduction in the number of degrees of freedom.

According to [8], bioinspired engineering or biologically inspired design is a paradigm for design innovation that uses parameters of Bionics, biomimetic, and biomimicry, in that all follow nature design and solutions for design ideas and optimization. The use of bioinspired solutions applied to engineering problems is a possibility of the development of new and innovative products with influence in several fields of engineering, including materials, energy, computing, robotics, biomedical, manufacturing, and others. For the project, bioinspiration can promote gains in reducing sensors and optimizing robotic and computational resources.

The application of bioinspired solutions, described in [9], has been divided into three stages:

- Identification of analogies with similar structures and methods;
- Detailed understanding and modeling of actual biological behavior;
- Engineering, which simplifies the model and adjusts for technical applications.

The bioinspired solutions applied in the robots are presented as possibilities for better use of hardware and software, promoting improvements in the systems. Examples for such improvements, presented in [10,11,12], are the development of ends for robotic arms in order to carry out the artificial pollination process, using stereo vision systems and sensors such as L.i.D.A.R. for the mapping of the environment and verification of the state of maturity of the flowers.

Bioinspired solutions applied in hardware and software for the control and optimization of the robots are employed in several works. Control of the omni-wheels robots using an algorithm bioinspired in the brain limbic system was developed in [13]. Algorithms of bioinspired optimizations, using particle swarm optimization and genetic algorithm, were used on the tune retinal models for hierarchical feature extraction of images [14]. In [15], a denoising algorithm was developed, bioinspired on bees for dim luminous conditions were used in the night vision algorithm to mimic the amplification of the transduction process in photoreceptors of Megalopta genalis. In [16], a bioinspired end in the bumblebee’s pollination mechanism attached to a robotic arm was developed, designed to carry out the pollination process.

Plantations are environments of continual change. The parts of the plants and flowers can grow, change color, or even fall off in a very short period, such as hours or days. Thus, autonomous robots that use image processing in the pollination process need an adjustable control method that does not depend on prior knowledge about the area to be pollinated.

Several uses of the positioning methods by sharpening in autofocus operators are based on Laplacian variance and histogram methods. A limitation of the methods is the previous calibration of the algorithm with object size data. For artificial pollination, image processing of the flower and the pollination environment requires a method that performs the convergence of the distance between the end of the pollination probe and the flower, without prior knowledge of the target size, that is robust to the amount of information, whereby filter information is unnecessary to the process, such as color, shape or number of objects. Several works developed bioinspired and unsupervised methods for robot control with a computational vision in object detection for indoor scene perception [17,18], navigation in open environments [19], and with robot network applications [20].

Using minimal amounts of cameras, especially in more simplified rovers, is a deter-mining factor for the miniaturization of platforms, with the recommendation of the use of unsupervised control methods with a single camera, with a low need for prior calibration in autofocus operators to calculate distances between objects.

Using autofocus operators is related to the focal length of the object as a function of the gradient of the objects at each point in the image. This generates even gradient variation functions, which need additional information about the object’s orientation relative to the focal point. A sensor that stores orientation information, and with a global coordinate system in relation to the environment, solves this limitation. Bees move long distances, detecting variations in the earth’s magnetic field in their abdomen. This sensitivity to the magnetic field can be used as an analogy with the solution of guidance for autofocus operators. An electronic compass is an efficient, low-cost sensor that can be used as a solution in the positioning process with autofocus operators.

In this paper, the development of an unsupervised repositioning method bioinspired on magnetic field memory of bees is proposed, with autofocus operators based on Laplacian entropy and variance, applied on pollination robots for precision agriculture. The method uses the calculation of the sharpness of different points of the image to verify the displacement between planes—probe and target. Besides the introduction, the paper is organized in three more sections, as follows. Section 2 presents the materials and methods used in the robot’s development and the repositioning method are presented. The results and discussions are presented in Section 3, and the final considerations drawn from this work are given in Section 4.

## 2. Materials and Methods

The probing robot operates with two image positioning systems. First, the real-time system uses a side camera that performs calculations based on the difference in sharpness between the acquired image planes, with the simultaneous acquisition of the rover’s geomagnetic orientation values. The lateral image of the environment allows the robot to visualize both the end of the probing system and the target.

Second, a computer vision system that uses the measurement of area in time and frequency. A system with LEDs and active markers was developed to perform the operation of autonomous lifting and vibration analysis by the image of the probe, from the images provided through the side camera of the probing system.

Both systems are real-time, operating at different times in the positioning process. Systems perform the main process of differentiating between target and probe tip through color, by angular information in the *hue-saturation-value* (HSV) system, a robust system implemented to the reconfiguration because of the variety of target types and the lack of a previous database.

The two systems act in a complementary way for the correct positioning of the probe. The first system is intended for angular positioning of the platform, resulting in the alignment between planes of the probe end and the target. The second positioning system also uses the side camera, but its purpose is to approach the target vertically, through the rotation of the probe end.

The proposed method applies to a mechanical system, bioinspired in the bee licking device, with gains in reducing moving parts and sensors to perform the pollination task. The positioning of the probe is bioinspired in the sensitivity of bees to the terrestrial magnetic field, with the use of a sensor, an electronic compass, and by the analysis of vibration of the distance in oscillatory systems.

The magnetic field information is stored in a terrestrial orientation vector, acquired in the scanning process. Through a search algorithm, the optimal magnetic orientation value that corresponds to the minimum difference in sharpness between the planes is recursively pursued, with the actuators being controlled in a closed-loop.

Image acquisition is performed through the side camera of the sounding system in a scanning process. The calculations of sharpness between different points of the same image are performed in parallel to the acquisition of a set of magnetic orientation values. This makes the method robust, reducing the cost in relation to stereo vision systems and the member of cameras as it improves the information extraction process between small nuances of the objects in the same image.

Issues such as the variation of shapes and quantities of objects of different statistics in a single image, the variation of luminosity and consequently variation of the gradient, and the need for calibration in each operating environment of the robot gave birth to the need of improved methods. The new method is requited to be less susceptible to the number of edges and more sensitive to the gradient found at the edges of the acquired image objects. Therefore, the use of entropy as a measure of average information found at the edges extracted by the Laplacian is one of the objects of the present study.

The selection of points of interest in the image is carried out through color, one of the main characteristics in relation to the target in agriculture. The selection of colors is carried out in the HSV color space, requiring a previous RGB-HSV transformation [21].

By observing some natural pollinating agents such as honeybees, interesting mechanisms are identified through the artificial pollination process, such as sensitivity and memory to the terrestrial magnetic field, which is a possible solution to the problem involving unsupervised positioning [22].

Magnetic field memory is an existing property in honeybees that allows them to navigate approximately twelve kilometers away from the hive; this capacity is used in the construction of the structures of their habitat at night, where their vision system has a loss of efficiency because of the lack of photons in the visible spectrum [22].

Complex scenarios, such as pollination, autonomous positioning in agricultural environments, precision positioning in microscopes, and lethal autonomous weapons systems, require the calculation of distances with every piece of information. However, the system must handle aggravating factors, such as distance to the target, atmospheric conditions in proximity, and the capacity to extract information only with the energy received from the target and photons in various frequencies. Using image acquisition systems and the subsequent extraction of all the necessary information about the target becomes more feasible, generating the need for algorithms capable of extracting small nuances such as the sharpness between close points in the same image [23].

The metrics for calculating sharpness found in the literature involve two general approaches:

- Autofocus operators used to calculate sharpness in a single image [24];
- Shape-from-focus are operators that calculate sharpness based on multiple images [25].

The Laplacian variance and the histogram entropy are some of the methods used to calculate sharpness in a single image [25].

A characteristic common to unsupervised positioning systems for precision agriculture is the need to calibrate the distances, shapes, and sizes of targets. Such tasks require robust algorithms to the unforeseen variations of these variables. In [26,27], a stereo kinematic method is used to compensate for parallax errors.

The implementation of the system proposed in this work is composed of three parts:

1. The development of an unsupervised repositioning method, bioinspired in the bees navigation system, which uses the sharpness and the magnetic field, by the Laplacian variance and entropy as autofocus operators;
2. Development of a complementary method of vertical positioning and vibration analysis by the image in time and frequency of the area between markers; and
3. The application of the solution in a probing robot bio-inspired in the bee licking system for precision agriculture—in this case, both the robot and the repositioning method are bio-inspired in the biological systems and behavior of bees.

### 2.1. Probing Robot Bioinspired in the Bee Licking System

The probing robot employed in this work is formed of a robotic platform with a differential locomotion system. They use brushed-type direct-current motors, coupled in gearboxes at the front side ends, with two castor wheels at the rear. The system is characterized by the use of two traction motors and four wheels, aided by a power supply system and a control signal to the DC motors, controlled by a pulse width modulation (PWM) pulse with a constant duty cycle. The probing system is composed of the structure, cable, and electromechanical system for collecting and supplying cable.

**Figure 1:**
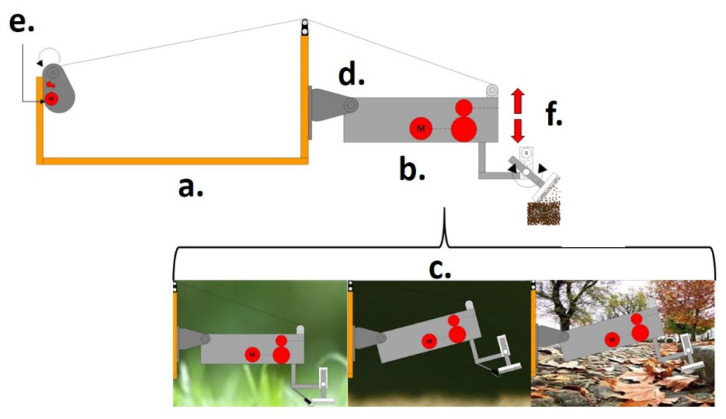
Schematic diagram of the platform’s locomotion system. (a) Mechanical structure; (b) Probe; (c) Sceneries of operation; (d) Knee-cap; (e) Electric motor; (f) Climb and descent movement.

Figure 1 shows the three-dimensional model, with:

- The traction forces on the front wheels that handle the translation and rotation of the platform;
- The lateral force is caused by the difference in rotation of the front wheels and their point of rotation;
- The kneecap point where the drilling system will rotate;
- Mechanical reduction mechanism is driven by an electric motor responsible for removing and supplying the cable that carries out the lifting of the platform.

**Figure 2:**
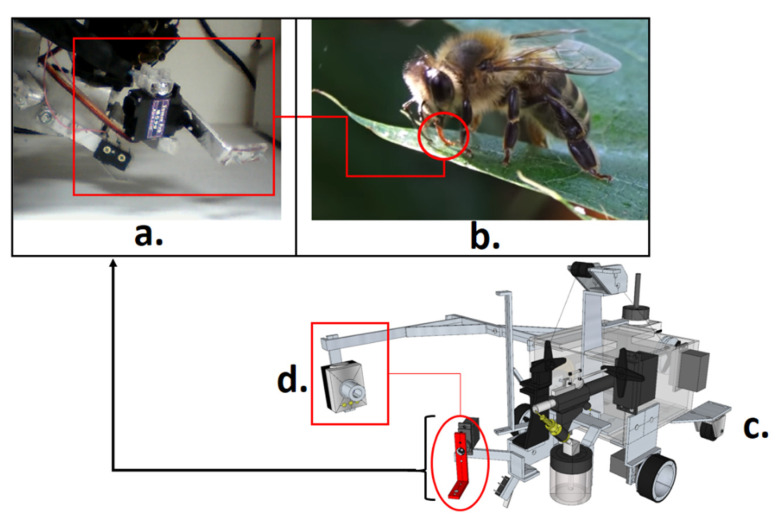
Rover of the bio-inspired probing system: (a) Probing system prototype, the image acquired by its side camera; (b) Licking system extracting liquids from a leaf; (c) Rover’s three-dimensional project including the side camera, probing system, and locomotion platform; (d) Side camera and the filmed Licking system model.

Figure 2 shows the three-dimensional model of the robotic platform (rover), with the bee-licking device, and the probing system model, with:

- A porous material support, responsible for direct contact with the sample to be removed from the flower;
- The lifting support, with the lifting cable;
- The actuator of the sprinkling system valve.

**Figure 3:**
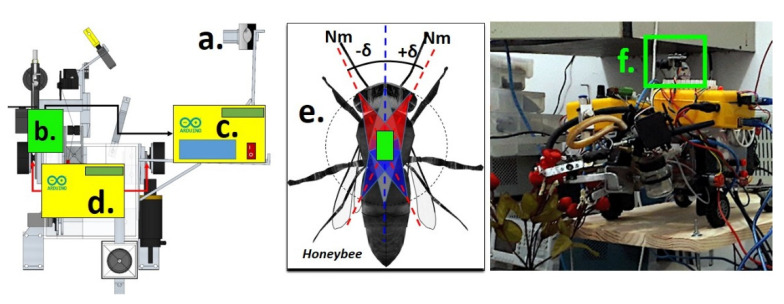
Bioinspired positioning method: (a) Lateral camera; (b) Geomagnetic sensor module; (c) Rover’s signal acquisition module; (d) Rover’s motion control module; (e) Illustration of the Magnetic detection through the abdomen of Honeybee in the green rectangle; (f) Geomagnetic sensor module implemented in rover.

Figure 3 shows the bioinspired repositioning method in bee geomagnetic field sensitivity, with the indications of the lateral camera (Figure 3a), i.e., acquisition of environment images, the orientations values of geomagnetic field by sensor module (Figure 3b), Rover’s signal acquisition module (Figure 3c), the rover’s motion control module that receives the signal from the image processing server (Figure 3d), an illustration of the magnetic detection through the abdomen of Honeybee that aids in the geomagnetic orientation process (Figure 3e), and the geomagnetic sensor module implemented in the rover (Figure 3f).

The post-processing that occurs in the method to extract color and sharpness characteristics of objects in different planes were associated with the geomagnetic orientation value of the electronic compass, for the subsequent search for the optimal geomagnetic angle, which has the smallest variation of sharpness between the object and target planes. As the scanning process is random, the magnetic declination values and environment variables will be entered as noise. An advantage of the described search method is not to use a previous database.

**Figure 4:**
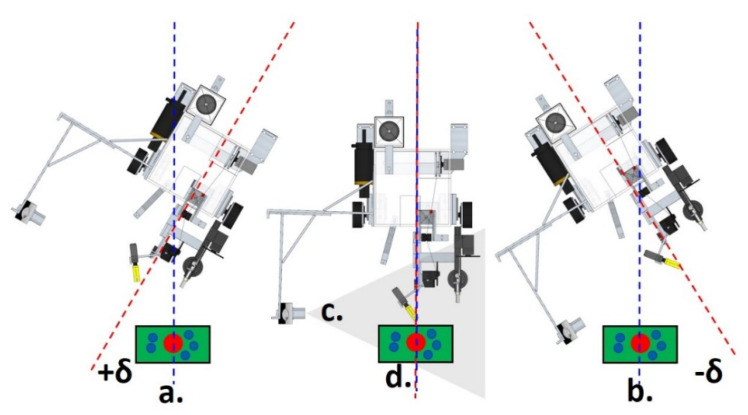
Implementation of the unsupervised method of the environment scanning process. (a) Sweep Angle +δ position; (b) Sweep Angle −δ position; (c) Side Camera; (d) Target and its possible locations.

Figure 4 shows the schematic diagram of the platform’s locomotion system, with the process of environmental scanning, image captures of objects with displaced planes, and focal lengths. Frames where the probing system and the object for which the probe must be repositioned are acquired with a side camera, type CMOS of 2.0 MP, installed in the Rover (Figure 4c), whose focus is adjusted, with resolution 30 FPS.

The electronic sensor for the acquisition of the magnetic orientation signal is the GY-273 geomagnetic sensor module with a 3-axis HMC5883L magneto-resistive sensor, and a resolution of 3 milli-Gauss. The sensor was installed in the center of rotation of the robotic platform, adjusted by the positioning algorithm, with the capacity to measure fields in the range between ± 8 Gauss, using the magnetic declination in São Luís- MA-BR on 8 April 2019, set to −20°53′ W.

The angle signal ranges from 0 to 359 degrees, with a minimum accuracy of 1 degree. The signal is sent through the I2C protocol to an ATmega328 micro-controller and transferred to the server through the serial protocol. After data processing by the server, the information on the platform direction and rotation angle is sent to an Arduino MEGA 250^®^ development board. The Arduino is coupled to L293D Integrated Circuit to control the direct-current motors responsible for the rotation of the platform.

### 2.2. Unsupervised Repositioning Bioinspired Method

The repositioning method is developed with two degrees of freedom: probe rotation and platform rotation. In the process, we considered the two angular variables controlled by a side camera and an electronic compass. This process uses the principle of the orientation of bees by terrestrial magnetic field memory.

Bees have particles sensitive to the magnetic field in their abdomen, which is why they can reorient themselves based on the earth’s magnetic field at the same without solar light presence, with the capacities used for spatial orientation outside and inside the hive during the pollination and construction processes [22].

The proposed bioinspired repositioning method uses two approximation hypotheses in conjunction with the orientation by the magnetic field, using only a camera and an electronic compass:

1. The area formed between active markers is extracted from the positions of their barycenters from the input image, relative to the positions of the extreme points of the probing system and the desired target;
2. Two objects in the same plane, or in the same image, have quantitative values very close to the sharpness. The difference between the measured sharpness of the objects is related to the distance between both planes. The size of this difference leads to control attitudes that seek a minimum difference value, representing the alignment between the target and the plane of the extremity probe.

Both hypotheses lead to an unsupervised method of approximation by image and magnetic orientation, a strategy similar to that used by bees in searching for food.

The method is robust to variations of the environment and objects that, in this case, are segmented by its color, i.e., the red-flower and yellow-end of the probe, which are default to both the desired hue for the probe end and the desired hue for the target, for which the probing system should be repositioned. Two processes are performed during the execution of the proposed method: environmental scanning and image captures.

In the scanning process, a random routine is generated with an angular variation in the clockwise and counterclockwise direction. Such a routine, whose range is limited to a fixed number of current pulses sent to direct current motors, in an open-loop regime and, because of imperfections of the mechanical, electrical, and floor systems under which the robot is located, the angles reached in each scanning routine are not symmetrical and have different modules.

The second process is the transformation of frame acquired from the RGB-HSV system for segmentation by color in the H plane, both at the end of the probe and the flower, with the aim of segmentation, binarization, and subsequent calculation of the barycenter of the object, probe, and flower. The two resulting images of the probe and the flower region are then converted to gray-scale. Then, they are convoluted with Laplacian kernels and their variances calculation is performed.

The resulting images are processed to get their respective probability density functions based on the normalization of the histograms, with the values of the variances and entropy of the Laplacian of these functions, both as measures of sharpness, of the probe and target. The statistics of the regions in the input image are then subtracted, which are later concatenated into two vectors.

The segmentation process uses transformation from the RGB system to the HSV system, selecting the polar range of color for targeting the desired region. The barycenters of the probe and the flower are calculated from the average of the coordinates of the pixels of the mask, resulting from the segmentation process in each range of the desired hue. Equation (1) presents transformation between RGB to HSV color systems.

(1) $$H\{\begin{array}{c} \begin{array}{cccc} 60\times \frac{G-B}{MAX-MIN}+0 & If\;MAX=R\; & \& & G\geq B\\ 60\times \frac{G-B}{MAX-MIN}+360 & If\;MAX=R & \& & G<B \end{array}\\ \begin{array}{cc} 60\times \frac{B-R}{MAX-MIN}+120 & If\;MAX=G\\ 60\times \frac{G-B}{MAX-MIN}+240 & If\;MAX=B \end{array} \end{array}\;\;,$$

where *H* is the polar hue value; *R*, *G*, *B* are the pixels values of matrixes in the input image on the color Cartesian System; and *MAX* and *MIN* are the respective maximum and minimum pixels values of the input image.

The results of the pixels histograms of the binarization process in the mask encourages the search for the horizontal and vertical coordinates of the barycenter by analyzing the lines and columns of the segmented region. An average of the pixel coordinates is performed, thus estimating the object’s barycenter that has the desired color. After the barycenter is found, the desired region is cut.

**Figure 5:**
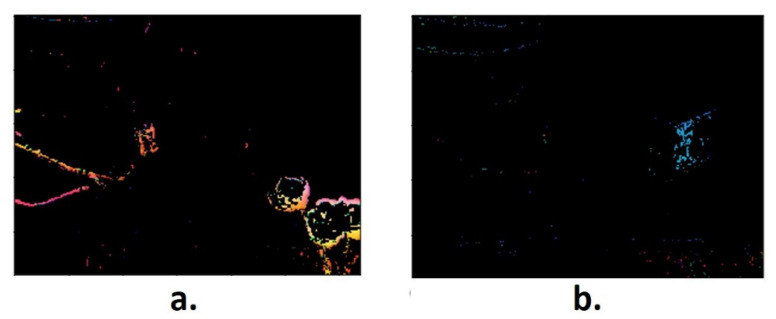
Mask of the interest Regions: (**a**) Target; (**b**) Probe end.

Figure 5 shows the masks related to the segmentation process of the probe tip and the target, the flower and region mask segmented based on red hue (Figure 5a), and a probe end region mask segmented based on yellow hue, whereby pixels are represented in blue in the mask (Figure 5b).

The barycenter of the objects is employed as central points for sampling the regions of interest, with a later transformation to gray-scale and the Laplacian operator for the extraction around the edges [13,14,15]. The matrixes resulting from the convolution process are vectored, stored, and used to calculate the variances, probability distributions, and entropies. Autofocus operator developed used as the first operation to extraction the Laplacian from the matrix or the image for the vectors *x* *e* *y* is characterized by:

(2) $$\;L_{x,y}=\frac{\partial^{2}I}{\partial x^{2}}+\frac{\partial^{2}I}{\partial y^{2}},$$

where $\;L_{x,y}$ corresponds to the Laplacian operator applied to the desired region of the image, $vec\left(\;L_{x,y}\right)$, is a one-dimensional vector of the vectorization operation in region of interest, and the image region probability vector is indicated by:

(3) $$H_{i}=-\sum_{i=1}^{i=len\left(Pr\right)}p_{i}\log p_{i},$$

where $H_{i}$ is the entropy of the segmented region in the respective frame *i*, $p_{i}$ is the element of the probability vector, with $i=len\left(P_{r}\right)$, the upper limit, and $P_{r}$ the last element of the probability vector.

From Equations (2) and (3), the search for the minimum element of the vector of differences, entropy, and variances of the Laplacian occurs. The index of the element of the difference vector is then compared to the index of magnetic orientation to find the optimal angle value to which the sounding system should be repositioned. Equation (4) shows the process of entropy subtraction of the respective regions, based on its Laplacian vectors.

(4) $$H_{dif}=H_{o}L_{probe}-H_{o}L_{target}\;,$$

where $H_{dif}$ is the vector generated by the differences between the entropy values of the probe $H_{o}L_{probe}$ and target $H_{o}L_{target}$, flower, generated for each image in the scanning process, see Figure 3.

The magnetic orientation angle value θ is synchronized to the frames, and models of the search for the magnetic orientation angle based on the smallest difference between entropies of the segmented regions is given by:

(5) $$Index_{\theta_{alvo}}\equiv Index_{H_{dif}^{*}\;},\;$$

where $Index_{\theta_{alvo}}$, the index in the magnetic orientation vector θ, has an angle value to which the probe should be repositioned, and $Index_{H_{dif}^{*}\;}$ is the optimal entropy vector element.

The magnetic reorientation process is performed in a closed loop based on the error e(t) and the proportional gain $K_{p}$, generating a control response u(t) transformed into a serial value and later sent to an ATMEGA 2560 micro-controller where the serial signal encoded in 1 byte with values 0–255 will be converted into pulses with adjustable polarity and duty cycle percentual (%DC).

Values between 0–127 activate the DC motor in a clockwise direction and values between 127–255 will activate the speed control of motors in a counterclockwise direction and DC control. For the mechanical reduction, the control action has its amplitude divided by a $K_{mec}$ factor, necessary for angular repositioning with a lower admitted error value $e{\left(t\right)}_{min}\leq 1{}^{\circ}$ for the repositioning operation. The magnetic reorientation sensor sends data via the I2C interface through an ATMEGA 328 micro-controller to the server where the image processing algorithm is executed, and the control signal is sent to the motors.

**Figure 6:**
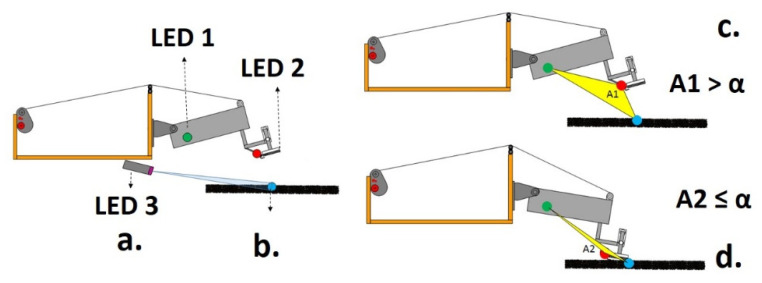
Markers diagram: (a) Marker locations; (b) Desired repositioning point; (c) Area A1; (d) Area A2.

Figure 6 shows the classification method by the threshold area, with the mechanical system diagram with markers. Feature extraction uses the area formed between the fixed frequency markers in the visible spectrum, implemented as the LEDs (Figure 6a). The desired repositioning point of the systems is visualized in Figure 6b; the A1 is the area between markers exceeding threshold α, probe distant from the desired point, and the A2 is the area between markers not exceeding threshold α, static probe at the desired point.

The different areas between the markers can be observed with the probe near and far from the desired positioning point (Figure 6b,c) with area calculation given by:

(6) $$A=\frac{1}{2}\|\det\left(M\right)\|\;,$$

where *A* is the area formed between the markers, and the matrix M formed by the average of the coordinates, barycenters of the maximum intensity pixels. The matrix M is given by:

(7) $$M=\left(\begin{array}{c} {\bar{x}}_{red}\;\;\;\;\;\;\;\;{\bar{y}}_{red}\;\;\;\;\;\;1\\ {\bar{x}}_{green}\;\;\;\;{\bar{y}}_{green}\;\;\;\;1\\ {\bar{x}}_{blue}\;\;\;\;\;\;\;{\bar{y}}_{blue}\;\;\;\;\;\;1 \end{array}\right)\;,\;$$

where $\bar{x}$ and $\bar{y}$ are the averages of the pixel coordinates of the maximum value in the respective red planes, green, blue.

From Equations (6) and (7) is possible to realize the classification criterion near the desired point, obtained by:

(8) $$C=\{\begin{array}{c} \;1,\;If\;A>\alpha \;\\ 0,\;If\;A\leq \alpha \; \end{array}$$

where α is the decision threshold, when the area between the markers exceeds the α threshold, the probe should be repositioned to the desired point (otherwise, a command will be generated to complete the repositioning process). *C* represents the operation class. Statement 0 represents static probe and statement 1, probe in lifting operation.

## 3. Results

**Figure 7:**
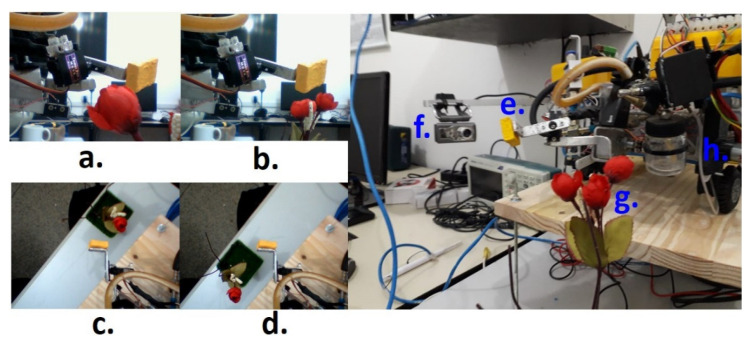
Prototype of the probing robot bio-inspired in the licking systems of the bees. (a) Distance from target to camera less than focal length; (b) Distance from target to camera greater than focal length; (c) Sweep angle −δ; (d) Sweep angle +δ; (e) Probe; (f) Lateral camera; (g) Flower or target; (h) Mechanical structure of the rover.

Figure 7 shows the prototype of the probing robot bioinspired in the licking systems of the bees, with the movement of the licking device (Figure 7a,d), indicating the probe (Figure 7e), lateral camera (Figure 7f), object target (Figure 7g), and the general mechanical structure of the rover (Figure 7h).

**Figure 8:**
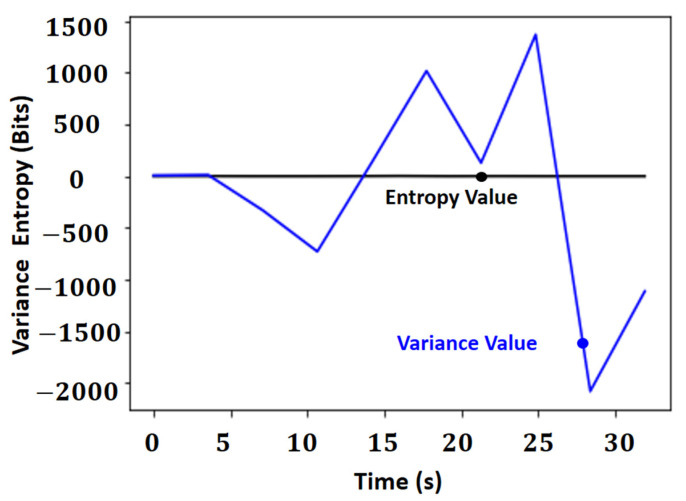
Comparison in the autofocus Laplacian’s entropy operator and the method of minimal differences based on Laplacian’s variance.

**Figure 9:**
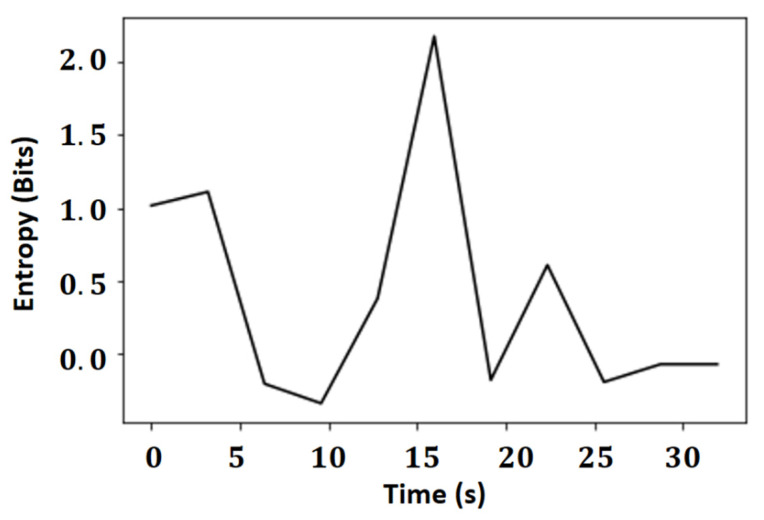
Vector of difference between entropies of the segmented regions.

Figure 8 shows the result of the autofocus operators based on the minimum difference between entropy and Laplacian variance in a positioning operation, and the vector of difference between entropies of the Laplacian in the regions can be seen in more detail in Figure 9.

The entropy value is on a much smaller scale than that of the variant, with a maximum variation of two bits, while the variance variations are greater than three thousand. Thus, it can be concluded that the entropy for this application offers results less susceptible to the number of edges of the image and more susceptible to the gradient of these edges, making the results more stable, given that entropy works with the amount of average information in the signal compared to the variance of the Laplacian and the autofocus operator used.

**Figure 10:**
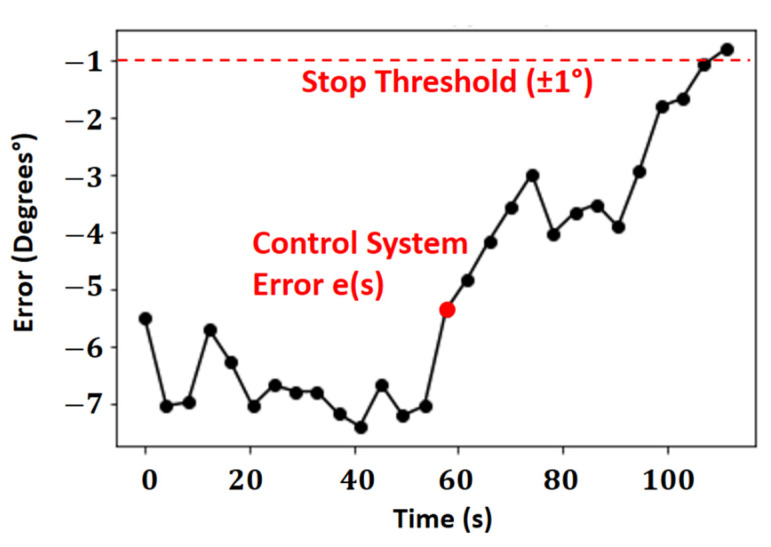
Control system of error in the repositioning operation.

The error control system in the repositioning operation, calculated by the entropy-based repositioning algorithm, is shown as a setpoint in Figure 10. The minimum error in degrees used as a threshold for stopping the repositioning system was ±1° (−1°≤e(t)≤1°). The error, calculated between the angle measured by the electronic compass and the setpoint, causes the repositioning to stop. From the angle relative, the smallest difference between the sharpness values and the limit at the edge of the object is calculated using the autofocus operator.

The autofocus system seeks an optimal situation, with the least absolute error in degrees. After the first scan of the objects, the system looks for the point of the lowest errors for the self-tuning effect, a value close to 1 degree. A second scanning is unnecessary, as the autofocus system will only use magnetic reorientation using the smallest error in the self-change process.

The movement of the probe is oriented by the signals of the probe and the marker corresponds to the angular movement, with a constant retraction speed and cable, with the oscillation spectrum of the markers, and the correlation with the distance between the kneecap and the end of the probe.

**Figure 11:**
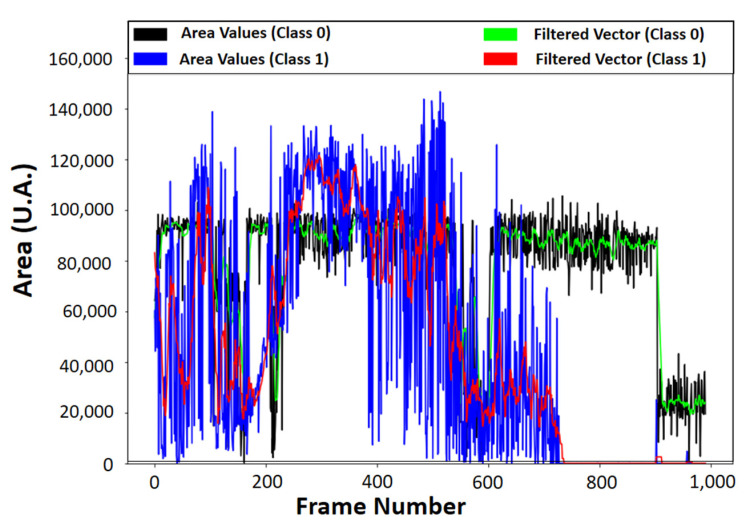
Calculated area values between the markers as a function of the frame number.

The Cartesian position of the markers has a non-linear shape, because of the angular positioning model, and images corresponding to class 1 show the probe lifting operation (see Figure 11). The signals correspond to the calculated areas with the probe in static operation, in the lifting operation, and the moving average filter applied.

**Figure 12:**
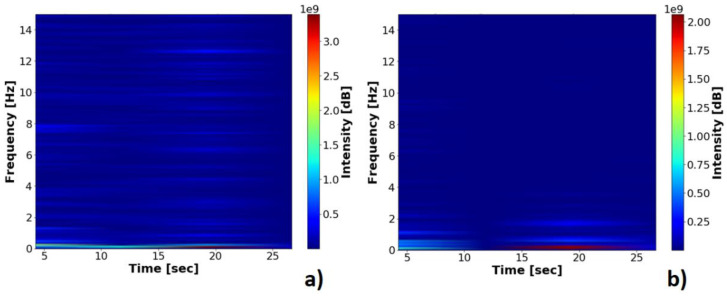
Spectrograms of the area vectors between the markers: (**a**) Probe in dynamic operation; (**b**) Probe in static operation.

The fast Fourier transform and the short-time Fourier transform applied to the vector composed of the values of the Area, in the static and dynamic operation spectrograms, along with the area signals plotted in Original and Filtered vectors, use a moving average filter with size window of ten units. The vectors of the area in the minimum position, class 0, and the lifting position, class 1, original and filtered, can be seen in Figure 11, and their spectrograms can be seen in Figure 12.

The normalized values of the light spectrum are submitted to Pearson’s correlation calculations between the spectrum of the area vector and the spectrum of the vertical and horizontal oscillation of each marker, to identify the source of the oscillation in the acquisition.

The classification by an area threshold method was used to analyze the time and frequency of oscillation of the points referring to the end of the probe and the kneecap point.

**Figure 13:**
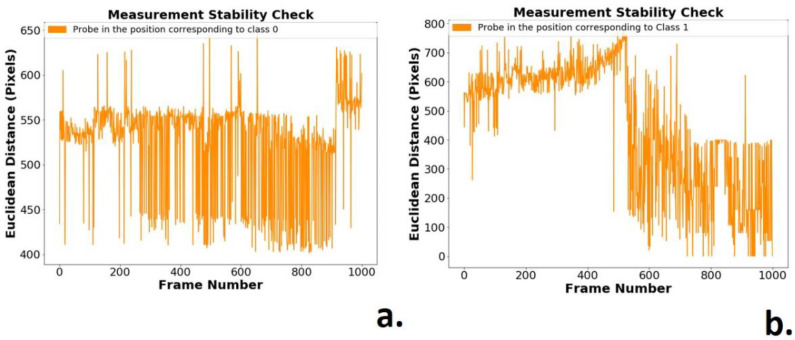
Euclidian distance between the red and blue markers: (**a**) Probe in static operation, class 0; (**b**) Probe in lifting operation, class 1.

Due to vibrations of the mechanical system and imperfections in the acquisition of images, the kneecap-end distance formed by the location of the barycenter can be treated as a time-variant vector (see Figure 13a,b). The measure of distance in pixels between the markers was then used as a measure of verification of variations in the image acquisition system.

**Figure 14:**
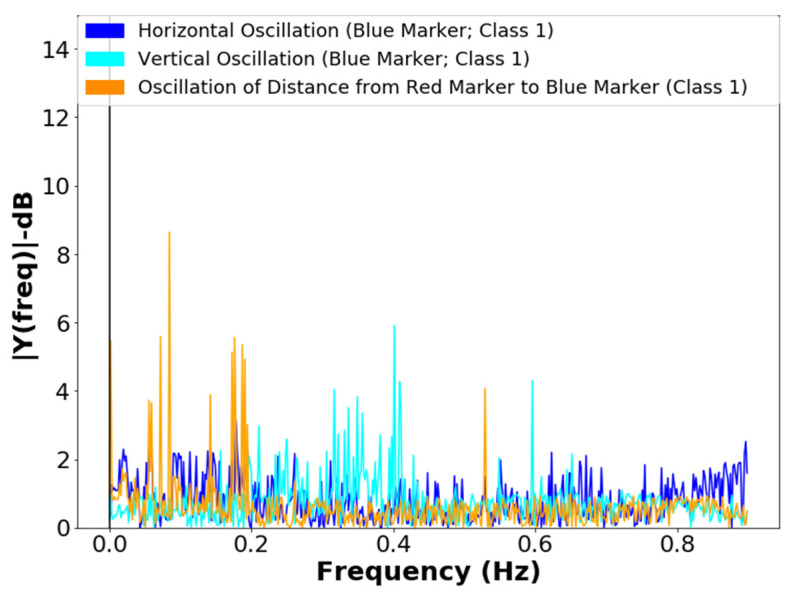
Frequency spectrum of the vertical and horizontal oscillations of the blue marker, in the lifting operation, class 1.

**Figure 15:**
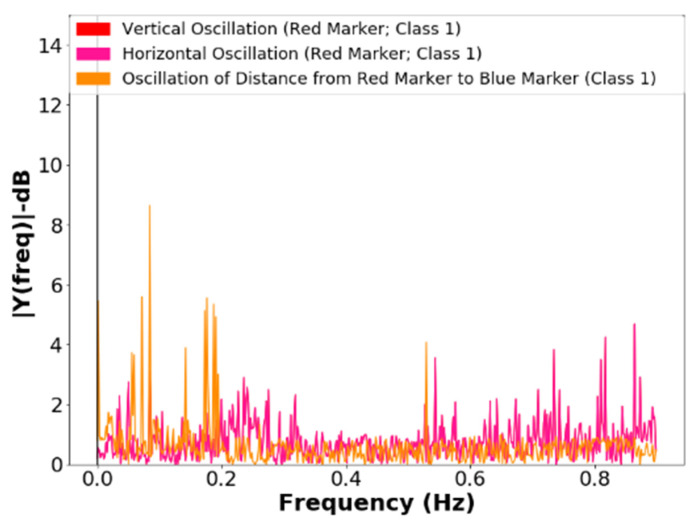
Frequency spectrum of the vertical and horizontal oscillations of the red marker, in the lifting operation, class 1.

The frequency analysis of the vertical and horizontal oscillations of the blue marker and the frequency analysis of the oscillations of the red marker in lifting operation, class 1, can be seen in Figure 14 and Figure 15. The lifting operation is the most critical in terms of failures and vibrations in the systems; the frequencies resulting from mechanical vibrations must also be observed. The proposal for a classifier requires a filter that tracks sharp transitions between area values with the aim of noise attenuation, considering the best statistical response of the signals for unsupervised repositioning.

The black line is the noise between the vector of the original area and the markers (class 0), and the blue line is the noise between the original area vector and the filtered vector with the probe in lifting operation (class 1). The instability of the area vectors, as observed in Figure 10, is associated with the variation of physical quantities, such as luminosity, mechanical vibrations absorbed by the camera, and noise in the acquisition process.

The developed algorithm performs two processes in the frames:

- The extraction of information in the noisy signal, with the frequency analysis of the positions of the markers, see Figure 12, Figure 13 and Figure 14;
- Verification of the correlations of the Euclidean distance between markers (Figure 12) and the position values of the barycenters of the patella markers (red marker) and of the probe tip (blue marker), whose values can be observed in Table 1 and Table 2.

**Figure 16:**
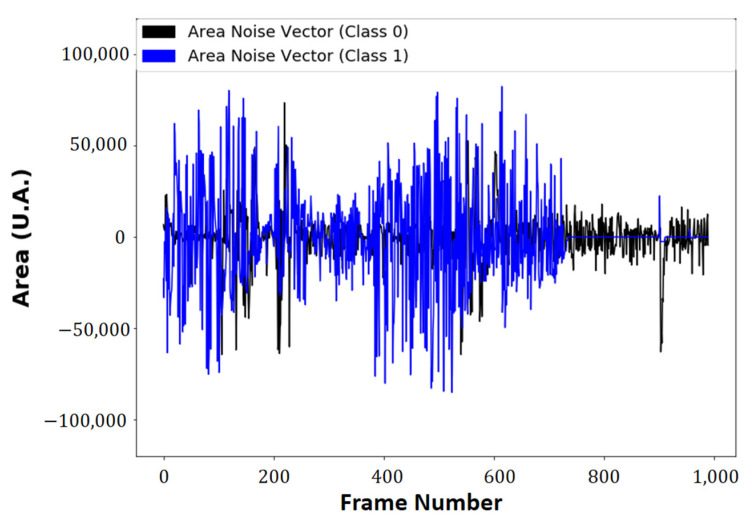
The noise of area vectors.

The resulting noise between the initial area vector is filtered, both in class 0 and in class 1 (Figure 16). The advantage of the developed method is the extraction of area information for an unforeseen noise.

**Figure 17:**
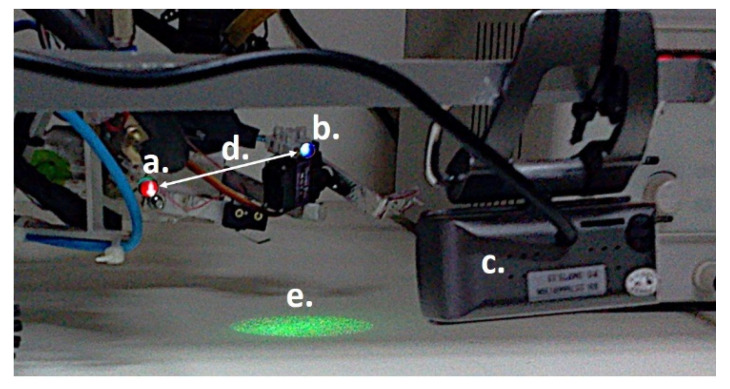
Markers used in frequency analysis: (a) Positions of the patella; (b) Position of the tip probe; (c) Lateral image of the camera; (d) Euclidean kneecap-tip distance; (e) The Target pointed by LED.

Figure 17 shows the positioning of the active markers, with:

- the LED red color inserted into positions of the patella, Figure 17a;
- the LED blue color inserted into the position of the tip, Figure 17b;
- the probing system inserted into the lateral image of the camera, Figure 17c;
- the target, identified by the green color, Figure 17e.

The vibration analysis and frequency correlation were performed on the kneecap and tip markers with the Euclidean kneecap-tip distance analysis, as seen in Figure 17d.

**Figure 18:**
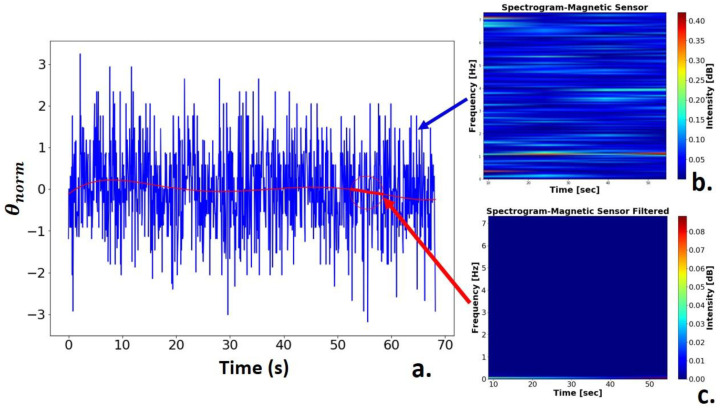
Geomagnetic signal: (**a**) Normalized geomagnetic signal; (**b**) Sensor noisy signal spectrogram; (**c**) Filtered signal spectrogram.

Figure 18 shows the result of the geomagnetic sensor signal treatment. The signal was normalized by applying statistical regression, which generated a fifth-degree polynomial, and was used to filter the information necessary for positioning (Figure 18a). From the results, the spectrograms of the normalized angle initial vector (Figure 18b) and the vector referring to the polynomial generated for filtering the signal frequencies (Figure 18c) were extracted.

Table 1 shows the Pearson correlation values between the oscillation of the patella and the tip markers and the distance to the tip patella (Figure 18d) used as a measure of stability in the process of acquisition. This measurement is performed both in static operation, with the probe stopped at the position of the minimum area between the markers, as in dynamic operation, and with the probe in operation. Table 2 shows the performed measurements with the probe, in constant speed lifting operation.

**Figure 19:**
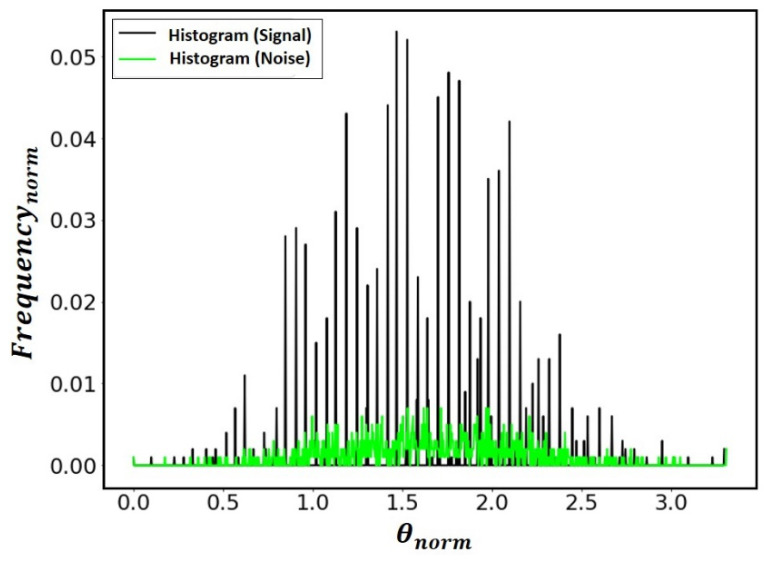
Normalized frequency histogram for estimating the probability distribution of the geomagnetic signal.

Figure 19 shows the histogram of the histograms of the system’s signal frequencies and noise, performed by estimating the probability distribution of the magnetic signal, used in the method developed for the rover. From the data on the frequency distribution of the signal and the noise, it is possible to identify changes in the statistical nature of the signal, enabling the configuration of specific filters for each type of noise.

From the results acquired, it can be evaluated that:

- The change in the positioning process because of the electromagnetic noise of the environment starts for angles (***θ****_error_* ≤ ±0.5°) performed in the tests and also because of the gaps in the mechanical systems, not converging in times less than 300ms and not generating repeatability, being susceptible to electromagnetic impulses from devices close to the place of operation.
- The system proved is satisfactory for presenting convergence to minimal angular error (***θ***_*error*(*min*)_ = ±1°), not requiring prior calibration about the target sizes. The color depth is used in the classification process with 8-Bit depth, applicable to commercial imaging systems.
- The convergence of the algorithm occurred within the proposal of the algorithm, which was to use the Laplacian entropy at the expense of the Laplacian variance as a new autofocus operator. Since Shannon’s entropy is the average information in a vector, the visible amplitude variation in relation to the Laplacian variance as an autofocus operator was used to overcome the problem of lack of prior information on the number of edges and shapes contained in the target region.
- The search algorithm used the magnetic field information is associated with the image of the probe tip in different planes, in relation to the target in the scanning process (δ ≈ ±30°).
- The search for the geomagnetic orientation that represented the smallest difference in sharpness between the end of the probe and the target neutralized the value of magnetic declination, which was affected by this variable only if, in the positioning process that lasts around three hundred seconds, disturbances occur geomagnetically or electromagnetics that effect an angle value change in the sensor relative to magnetic north of (±1°).

## 4. Final Considerations

The development of an unsupervised repositioning method bio-inspired by a magnetic field memory of the bees for application in pollination was presented in this work. The method uses the terrestrial magnetic field, and the sharpness, by Laplacian variance and entropy, of the images segmented by color in the HSV system in real time. The project employed a bio-inspired robot in the licking system of the bees. From the results, it was observed that, among the oscillations of the Euclidean distance, the Pearson correlation was higher for the extremity marker, with a greater reduction in frequencies above 0.8 Hz, showing that the spectra are more sensitive to the variation of the marker of the extremity, and that the method is not sensitive to disturbances caused by vibrations in image acquisition systems. For tiny areas (<105 A.U.–Class 0), the classification can be performed considering the frequencies present because of vibrations caused by mechanical imperfections and electrical noises in the acquisition system, without loss of information and with system self-adjustment. The system proved to be satisfactory for presenting convergence to minimal angular error (***θ***_*error*(*min*)_ = ±1°) without requiring prior calibration for the target sizes or the color depth used in the classification process with 8-Bit depth, which is applicable to commercial imaging systems.

## Figures and Tables

**Table 1:** Pearson correlation values between markers and the kneecap to extremity distance with static probe in minimal position.

Position of Markers	Oscillation Orientation	Value
Knee-Cap (Red Marker)	Horizontal	0.04089471
Knee-Cap (Red Marker)	Vertical	0.04089471
Extremity (Blue Marker)	Horizontal	−0.12295771
Extremity (Blue Marker)	Vertical	−0.14011202

**Table 2:** Pearson correlation values between the markers and the kneecap to extremity distance with the probe in lifting operation.

Position of Marker	Oscillation Orientation	Value
Kneecap (Red Marker)	Horizontal	0.05307159
Kneecap (Red Marker)	Vertical	0.05307159
Extremity (Blue Marker)	Horizontal	−0.05106755
Extremity (Blue Marker)	Vertical	0.29337411

## References

[1] United Nations Population. https://www.un.org/en/sections/issues-depth/population.

[2] Pedersen S.M., Lind K.M. (2017). Precision Agriculture: Technology and Economic Perspectives.

[3] Jawad H.M., Nordin R., Gharghan S.K., Jawad A.M., Ismail M. (2017). Energy-Efficient Wireless Sensor Networks for Precision Agriculture: A Review. Sensors.

[4] Ivanov S., Bhargava K., Donnelly W. (2015). Precision Farming: Sensor Analytics. IEEE Intell. Syst..

[5] Zhang Q. (2016). Precision Agriculture Technology for Crop. Farming.

[6] O’Toole C., Raw A. (2004). Bees of the World.

[7] Lal R., Stewart B.A. (2016). Soil-Specific Farming: Precision Agriculture.

[8] Silva Júnior P.F., Silva P.H.d.F., Serres A.J.R., Silva J.C., Freire R.C.S. (2016). Bio-inspired design of directional leaf-shaped printed monopole antennas for 4G 700 MHz band. Microw. Opt. Technol. Lett..

[9] Dressler F., Ozgur B.A. (2010). A Survey on Bio-inspired Networking. Comput. Netw..

[10] Shaneyfelt T., Jamshidi M.M., Agaian S. (2013). A vision feedback robotic docking crane system with aplication to vanilla Pollination. Int. J. Autom. Control..

[11] Yuan T., Zhang S., Sheng X., Wang D., Gong Y., Li W. An Autonomous Pollination Robot for Hormone Treatment of Tomato Flower in Greenhouse. Proceedings of the 2016 3rd International Conference on Systems and Informatics (ICSAI).

[12] Brunelli D., Tosato P., Rossi M. (2016). Flora Health Wireless Monitoring with Plant-Microbial Fuel Cell. Procedia Eng..

[13] Lamini C., Benhlima S., Elbekri A. (2018). Genetic Algorithm Based Approach for Autonomous Mobile Robot Path Planning. Sci. Direct.

[14] Crespo-Cano R., Cuenca-Asensi S., Fernández E., Martínez-Álvarez A. (2019). Metaheuristic Optimisation Algorithms for Tuning a Bioinspired Retinal Model. Sensors.

[15] Hartbauer H. (2020). From Insect Vision to a Novel Bio-Inspired Algorithm for Image Denoising. Biomimetics.

[16] Ohi N., Lassak K., Watson R., Strader J., Du Y., Yang C., Hedrick G., Nguyen J., Harper S., Reynolds D. Design of an Autonomous Precision Pollination Robot. Proceedings of the 2018 IEEE/RSJ International Conference on Intelligent Robots and System (IROS 2018).

[17] Wang L., Li R., Sun J., Liu X., Zhao L., Seah H.S., Quah C.K., Tandianus B. (2019). Multi-View Fusion-Based 3D Object Detection for Robot Indoor Scene Perception. Sensors.

[18] Zhou X., Bai T., Gao Y., Han Y. (2019). Vision-Based Robot Navigation through Combining Unsupervised Learning and Hierarchical Reinforcement Learning. Sensors.

[19] Lu K., Li J., An X., He H. (2015). Vision Sensor-Based Road Detection for Field Robot Navigation. Sensors.

[20] Fraga D., Gutiérrez Á., Vallejo J.C., Campo A., Bankovic Z. (2011). Improving Social Odometry Robot Networks with Distributed Reputation Systems for Collaborative Purposes. Sensors.

[21] Woods R.E., Gonzalez R.C. (2007). Digital Image Processing.

[22] Liang C.-H., Chuang C.-L., Jiang J.-A., Yang E.-C. (2016). Magnetic Sensing through the Abdomen of the Honey bee. Sci. Rep..

[23] De K., Masilamani V. (2013). Image Sharpness Measure for Blurred Images in Frequency Domain. Procedia Eng..

[24] Mahmood U.A. (2018). Analysis of Blur Measure Operators for Single Image Blur Segmentation. Appl. Sci..

[25] Pertuz S., Puig D., Garcia M.A. (2012). Analysis of focus measure operators for shape-from-focus. Pattern Recognit..

[26] Elstone L., How K.Y., Brodie S., Ghazali M.Z., Heath W.P., Grieve B. (2020). High Speed Crop and Weed Identification in Lettuce Fields for Precision Weeding. Sensors.

[27] Zhang T., Huang Z., You W., Lin J., Tang X., Huang H. (2020). An Autonomous Fruit and Vegetable Harvester with a Low-Cost Gripper Using a 3D Sensor. Sensors.

